# A Hand-book of Medical Electricity

**Published:** 1873-08

**Authors:** 


*A Handbook of Medical Electricity.* By Herbert Tibbits, M. D., L. R. C. P., London. With sixty-four illustrations. Philadelphia: Lindsay & Blakiston, 1873.

This little book is published not with the expectation of presenting any new ideas on the subject of electricity, or of controverting old theories. It was written in order to place in the hands of the general practitioner a guide to the application of electricity which would make its use both satisfactory to the physician and beneficial to the patient. The author, as medical superintendent of a hospital for paralyzed and epileptics, has had abundant opportunity of observing the value of electricity as a therapeutical agent. His observations are carefully made and his conclusions stated in simple language. The nomenclature adopted by the author is briefly explained in the opening pages which will materially assist the reader in the study of the work; next follows a consideration of the instruments which the author has found to be of most service, and whose price does not place them beyond reach of all.

The author gives in conclusion some simple rules for the application of electricity, which are admirably plain and uncomplicated, and will materially assist the practitioner in the use of electricity. Our own experience in the use of this agent has been limited and unsatisfactory, and from what we have seen of its use in the hands of others, we have been unable to draw any very encouraging conclusions, this may, however, be the result of a lack of knowledge concerning its proper application, and we are far from condemning its use or of doubting its utility in the large number of cases placed on record by those skilled in its practical application. Should other cases present themselves wherein the use of electricity would seem to offer any inducements, with the directions of the author before us its application could be made in a systematic manner.

The book is presented in a pleasing and convenient form, and is printed in large, clear type; the illustrations are probably intended to enable the reader better to appreciate the teachings of the author, but were the text no clearer an exponent of the author's views than are the illustrations, we fear the reader would be left in the dark indeed. For those in search of a practical handbook in electricity we have no hesitation in recommending the one under consideration.

